# Factors associated with wife beating in Egypt: Analysis of two surveys (1995 and 2005)

**DOI:** 10.1186/1472-6874-8-15

**Published:** 2008-09-18

**Authors:** Manas K Akmatov, Rafael T Mikolajczyk, Shokria Labeeb, Enas Dhaher, Md Mobarak Khan

**Affiliations:** 1 Department of Public Health Medicine, School of Public Health, University of Bielefeld, Bielefeld, Germany; 2 Faculty of Nursing, University of Assiut, Assiut, Egypt

## Abstract

**Background:**

Wife beating is an important public health problem in many developing countries. We assessed the rates of wife beating and examined factors associated with wife beating in 1995 and 2005 in Egypt.

**Methods:**

We used data from two Demographic and Health Surveys (DHS) conducted in Egypt in 1995 and 2005 using multistage household sampling. Data related to wife beating included information from 7122 women in 1995 and 5612 women in 2005. Logistic regression was used to analyze factors independently associated with wife beating. Special weights were used to obtain nationally representative estimates.

**Results:**

In 1995 17.5% of married women in Egypt experienced wife beating in the last 12 months, in 2005 – 18.9% or 16.0%, using different measures. The association between socio-demographic differentials and wife beating was weaker in the newer survey. The 12-month prevalence of wife beating was lower only when both partners were educated, but the differences across education levels were less pronounced in 2005. Based on the information available in the 2005 survey, more educated women experienced less severe forms of wife beating than less educated women.

**Conclusion:**

Different measures used in both surveys make a direct comparison difficult. The observed patterns indicate that the changes in prevalence may be masked by two opposite processes occurring in the society: a decrease in (severe forms of) wife beating and an increase in reporting of wife beating. Improving the access to education for women and raising education levels in the whole society may help reducing wife beating.

## Background

Intimate partner violence is a worldwide problem, present in all cultures and societies [1]. The rates of wife beating are higher among young couples, in poor and less educated, but beating occurs in all socio-economic groups and also across more mature couples [2,3]. The prevalence of wife abuse and battering has been studied extensively in many societies including Arabic countries [4-7]. Consistent with the high acceptance of wife beating, surveys in Egypt, Palestine, and Tunisia indicated a very high prevalence of wife beating in these countries [8]. Egypt is a country with both a high prevalence of wife beating compared to other countries [9,10] and an exceptionally high level of approval regarding the husbands' right to wife beating [11,12]. For instance, almost 9 in 10 ever-married women accept at least one reason for wife beating in Egypt [12]. Many activities to educate women and improve the situation in Egypt, including an increased availability of shelters may have affected the attitude towards the acceptability of beating [13]. Also a new divorce law was introduced in 2000 [14]. While this law still makes the divorce difficult, it was an improvement towards previous status.

Two Demographic and Health Surveys (DHS) conducted 10 years apart (in 1995 and 2005) in Egypt obtained information about wife beating [11,15]. In these two surveys different methodologies were used to assess wife beating. In 1995, DHS introduced for the first time a module assessing the status of woman in the country including a single non-standardized question on wife beating [9]. This was the first attempt to collect information on wife beating in the history of DHS. In 2005, a standardized approach of measuring wife beating was used, which is based on 8 items describing different forms of violence. While comparing the information collected in the surveys three different underlying mechanisms of the observed difference should be considered: an impact of the different way the question was asked, a change in prevalence of wife beating over time and/or a change in reporting of wife beating. We use the data of these surveys to a) assess the rates of wife beating in 1995 and 2005, and b) examine factors associated with wife beating in the two surveys. Additionally, we assess more in depth the association between education of both partners and the patterns of violence.

## Methods

### Sample

Several DHS were conducted in Egypt, but only two of them investigated issues surrounding wife beating: the surveys conducted in 1995 and 2005 (further: EDHS-1995 and EDHS-2005). Both EDHS were nationally representative household surveys applying multistage sampling technique [11,15]. DHS's all over the world apply a standard questionnaire with several modules covering information about reproductive health of women such as contraceptive knowledge and use, fertility preferences and attitudes about family planning, pregnancy outcomes, breastfeeding and socio-economic characteristics [16]. EDHS-1995 was started in January 1995 and finished in December 1995. EDHS-2005 lasted from February to July 2005. Both surveys conducted household interviews using standardized questionnaire in Arabic language. In both survey the response rates were very high (about 99%). In EDHS-1995 an additional module was included to collect information regarding the women's status in households, including information about wife beating. This was done in one-third of all households selected for EDHS-1995 (n = 7122). The module was used for the first time in Egypt by DHS [9]. In EDHS-2005 information about wife beating was obtained for a randomly selected subsample for the anaemia testing (n = 5612). The 2005 survey used standardized questions based on guidelines from the World Health Organization and were first implemented in the DHS in Nicaragua in 1998 [9].

### Outcome variables

The outcome variable in our analysis: beaten by husband in the last 12 months (yes/no) was created from several questions, asked in slightly different ways in the two surveys. In EDHS-1995 the questions were "From the time you were married has anyone ever beaten you?", "Can you tell me who has done this to you since you were married?" and "Approximately, how many times were you beaten [by this person] in the past one year?". Women who answered positively on the first question, named their husband as the person doing the beating in the second question, and reported being beaten by husband once or more in the last 12 months were classified into the "yes" group. All remaining women were classified as "no". In EDHS-2005 several questions regarding the type of beating were asked, namely "Does/did" your (last) husband ever push you, shake you, or throw something at you; slap you or twist your arm; punch you with his fist or with something that could hurt you; kick you or drag you; try to strangle you or burn you; threaten you with a knife, gun, or other type of weapon; attack you with a knife, gun, or other type of weapon; physically force you to have sexual intercourse with him when you did not want to?" and "How often did this happen during the last 12 months?". Women who gave a positive answer for any type of beating and reported that it happened once or more in the last 12 months were classified as "yes", all remaining women as "no" – this is used as standard definition in the further text. We also used a more restricted definition of wife beating in the 2005 survey, based only on 6 items: slap you or twist your arm; punch you with his fist or with something that could hurt you; kick you or drag you; try to strangle you or burn you; threaten you with a knife, gun, or other type of weapon; attack you with a knife, gun, or other type of weapon.

### Socio-demographic variables

We analysed the association between several socio-demographic factors such as age, age at first marriage, place of residence (urban vs. rural), region, educational level of respondent and partner, religion, current working status and wife beating. We also included the variable blood relationship to husband (with two categories "yes" and "no") in the analysis, since beating occurs more frequently in partnerships with blood relationship [17]. The original variable consisted of seven categories for different degrees of blood relationship; any type of blood relationship was classified as yes.

### Statistical analysis

Chi-square test was used to analyse bivariate associations between wife beating and the other variables. In a second step, all considered variables were included jointly in a multivariable logistic regression model for each survey separately. Prior to analysis we ruled out a multicollinearity between independent variables using methods described by Allison [18].

The simultaneous associations of the education of both spouses with the outcome were investigated in a separate analysis. The model included education of each of the spouses, interaction between them, the indicator variable coding the difference between both surveys and the interaction between education and the indicator variable. In this way we could investigate whether education of the other partner had a modifying effect and whether the effects of education and their interaction were different in the two survey rounds. This model was reduced by removing terms for which the Wald-test indicated no significance and from the final model the proportion of women experiencing wife beating was calculated for different education categories. For this analysis the four education categories for the respondent and her husband were each collapsed into two: low = no/primary education and high = secondary/higher education.

In the next step we aimed to classify women according to the kind of wife beating they are experiencing. Since the EDHS-2005 investigated 8 different types of wife beating and some of them were highly correlated, we performed first a factor analysis to find underlying patterns of violence. The obtained factors were used to define clusters of women experiencing different forms of violence. Then the association between forms of experienced violence and education level of the women was investigated.

In all analyses, special weights (included in the dataset) were used to obtain nationally representative estimates. Data analysis was performed using the statistical program SAS for Windows, version 9.1.

## Results

### Description of the sample

In general, some positive tendencies can be observed over the period of ten years between the two EDHS (Table 1): The education level of the women and their partners increased substantially; the percentage of women with no education decreased by 10 percent and the percentage of women with higher education increased by 4 percent. The same was observed for their partners; the percentage of men with no education decreased by 8 percent and the percentage of highly educated men increased by 4 percent. Smaller differences were observed for the current working status of women. The percentages of women with a very young age at marriage or at birth of their first child were substantially reduced. Also a considerably lower percentage of marriages between related individuals (with a blood relationship) was observed in the newer survey round.

**Table 1:** Characteristics of the samples from the two Egypt demographic and health surveys (percent do not add to 100 due to rounding errors)

Variables	EDHS-1995 (n = 7122)	EDHS-2005 (n = 5612)
	Percent	Percent

Region		
Urban Governorates	22.8	16.6
Lower Egypt – Urban	12.9	11.9
Lower Egypt – Rural	29.8	31.9
Upper Egypt – Urban	10.2	12.5
Upper Egypt – Rural	23.4	26.0
Frontier Governorate	0.9	1.2

Age		

15–19	4.6	4.5
20–24	14.5	14.6
25–29	18.3	18.7
30–34	17.8	16.9
35–39	18.1	16.6
40–44	13.7	14.7
45–49	13.0	14.0

Education		

No education	44.3	34.3
Primary	25.2	15.8
Secondary	23.8	38.8
Higher	6.8	11.1

Partner's education		

No education	31.3	23.4
Primary	27.7	19.2
Secondary	29.6	41.9
Higher	11.5	15.6

Religion		

Muslim	94.9	94.9
Christian	5.2	5.2

Current working status		

Yes	18.5	21.6
No	81.5	78.4

Age at marriage		

<= 19	61.9	53.9
20–30	37.2	44.6
>= 31	0.9	1.5

Number of children in a household		

0–1	21.7	23.7
2–5	56.1	63.0
>5	22.2	13.4

Blood/marriage relationship to husband		

Yes	42.3	33.2
No	57.7	66.8

### 12 month prevalence and factors associated with wife beating

The prevalence of wife beating in the last 12 months was 17.5% in 1995 and 18.9% in 2005, based on the single question in 1995 and all items used in 2005. When in the 2005 survey wife beating was defined only based on the items clearly referring to beating the resulting prevalence was 16%. The most frequent forms of wife beating were pushing and slapping (Table 2).

**Table 2:** Rates of different type of wife-beating in the 2005 DHS survey in the last 12 months

Does/did your husband:	%
push you, shake you, or throw something at you?	14.1
slap you or twist your arm?	14.9
punch you with his fist or with something that could hurt you?	7.0
kick you or drag you?	3.2
try to strangle you or burn you?	0.5
threaten you with a knife, gun, or other type of weapon?	0.3
attack you with a knife, gun, or other type of weapon?	0.1
physically force you to have sexual intercourse with him when you did not want to?	3.9

The bivariate analysis indicates that the proportion of beaten women was lower in urban regions than in rural in both survey years (Table 3, 2nd and 4th column). The proportion of women who experienced beating was considerably higher among women with no education compared to those with higher education in both surveys. The same trend was observed for partner's education. Wife beating decreased with age of the respondent, but was higher for women who married and had first birth at a young age.

**Table 3:** Prevalence of wife beating in the last 12 months by different characteristics and factors associated with wife beating in multiple logistic regression (nationally representative results using special weights)

	EDHS-1995		EDHS-2005	
	Proportion of beaten respondents (%)	Adjusted Odds ratio* (CI 95%)	Proportion of beaten respondents (%)	Adjusted Odds ratio* (CI 95%)

Region				
Lower Egypt – Urban	14.2	1.01 (0.79–1.28)	15.7	0.97 (0.73–1.29)
Lower Egypt – Rural	19.7	1.16 (0.96–1.40)	22.5	1.16 (0.93–1.45)
Upper Egypt – Urban	19.3	1.38 (1.09–1.77)	15.6	0.86 (0.65–1.14)
Upper Egypt – Rural	21.3	1.17 (0.95–1.44)	19.7	0.79 (0.62–1.01)
Frontier Governorate	8.4	0.51 (0.21–1.28)	14.2	0.72 (0.35–1.50)
Urban Governorates	13.8	1	15.6	1

Education				

Higher	4.0	0.30 (0.17–0.53)	7.0	0.29 (0.19–0.44)
Secondary	12.3	0.56 (0.45–0.70)	17.1	0.63 (0.51–0.77)
Primary	21.5	1.10 (0.95–1.29)	23.0	1.12 (0.92–1.38)
No education	20.9	1	22.9	1

Age of respondent				

15–19	24.9	5.24 (3.46–7.93)	15.9	2.50 (1.54–4.06)
20–24	24.2	4.57 (3.34–6.24)	23.0	3.41 (2.47–4.71)
25–29	20.9	3.34 (2.51–4.45)	21.0	2.69 (2.01–3.60)
30–34	17.9	2.65 (2.00–3.51)	22.2	2.57 (1.94–3.40)
35–39	17.7	2.39 (1.81–3.14)	17.9	1.72 (1.30–2.28)
40–44	13.1	1.65 (1.23–2.22)	15.8	1.37 (1.02–1.82)
45–49	9.4	1	13.1	1

Religion				

Muslim	18.2	1.49 (1.06–2.09)	19.2	1.26 (0.88–1.79)
Christian	11.5	1	13.4	1

Current working status				

No	19.1	1.14 (0.94–1.39)	19.4	0.99 (0.82–1.19)
Yes	12.5	1	16.7	1

Age at first marriage				

<= 19	19.5	0.83 (0.36–1.91)	21.7	0.94 (0.44–2.01)
20–30	15.3	1.04 (0.45–2.39)	15.8	0.95 (0.45–2.00)
>= 31	13.3		9.7	

Number of children in a household				

0–1	18.6	0.79 (0.60–1.02)	14.6	0.54 (0.39–0.74)
2–5	18.3	1.02 (0.85–1.23)	20.4	0.94 (0.75–1.18)
>5	16.3	1	19.3	1

Blood/marriage relationship to husband				

No	18.5	1.26 (1.11–1.44)	18.6	1.07 (0.92–1.25)
Yes	17.1	1	19.4	1

Partner's education				

Higher	5.4	0.41 (0.27–0.60)	10.0	0.64 (0.46–0.89)
Secondary	15.8	0.75 (0.62–0.91)	19.5	0.81 (0.66–0.98)
Primary	22.1	1.02 (0.87–1.19)	18.2	0.63 (0.51–0.78)
No education	20.7	1	24.4	1

* Adjusted for all other variables in the table

In multivariable logistic regression, all differences between categories of the above mentioned variables in regard to wife beating were reduced (Table 3, 3rd and 5th column). The estimates for education in bivariate and multivariable analysis were similar for a woman's education but not for her partner's education. We observed minor differences when the analysis was repeated with the outcome variable based on restricted items (data not shown).

In regard to differences in factors between the two EDHS rounds associated with wife beating, we found significant results for the education of the partner, the interaction between a woman's and her partner's education, region of the country and age at first marriage (data not shown). In 2005 the decrease in wife beating with increasing level of partner's education was less pronounced. Similarly, the difference between regions was reduced as compared to 1995. In regard to the age at first marriage, the difference occurred only in the group marrying at age 30 plus years: such women had a reduced risk for wife beating in 1995 and increased risk in 2005. The analysis repeated with the outcome variable based on restricted items displayed only minor differences (data not shown).

When the effects of a woman's and her partner's education were both taken into account, the prevalence of wife beating was reduced only when both partners had secondary or higher education (Table 4). The extent of this reduction was lower in 2005 in comparison to 1995.

**Table 4:** Female and male education and the prevalence of wife beating (nationally representative estimates using special weights)

		EDHS-1995		EDHS-2005	
Female's education	Male's education	Respondents in this category (%)	Of these experiencing wife beating (%, 95% CI)	Respondents in this category (%)	Of these experiencing wife beating (%, 95% CI)

No and primary	No and primary	59	22 (21–23)	35	21 (19–23)
No and primary	Secondary and higher	16	19 (17–21)	14	27 (24–30)
Secondary and higher	No and primary	4	18 (13–23)	7	23 (19–27)
Secondary and higher	Secondary and higher	22	9 (8–10)	45	14 (13–15)

### Patterns of violence

**Figure 1:**
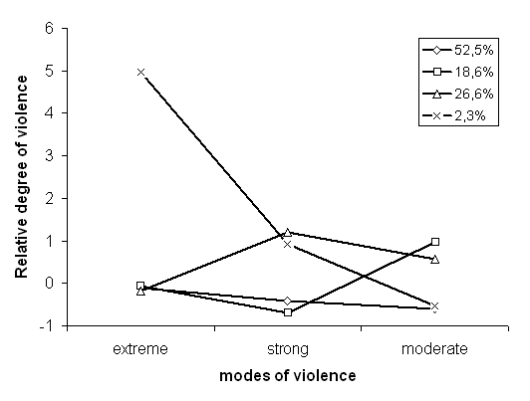
**Patterns of wife beating and proportion of women within clusters defined by these patterns in 2005 survey** (only women who experienced wife beating within last 12 months) (on the y-axis score indicating average values of the given group relative to the remaining population – 0 is the median score in the total sample).

Factor analysis revealed three underlying forms of violence: extreme (items: strangled/burned, threatened with weapon, attacked with weapon), strong (items: kicked/dragged, punched) and moderate (items: pushed, slapped/twisted, not forced sexually). Women who experienced violence in the past 12 months clustered in four groups which can be defined by patterns of violence (Figure 1). The smallest group (2.3%) experienced extreme forms of violence; the largest group (52.5%) scored low on all three forms of violence; 18.6% experienced mainly moderate forms of violence and 26.6% experienced strong and moderate forms of violence.

## Discussion

Information from two DHS conducted 10 years apart in Egypt was evaluated to investigate the prevalence of and factors associated with wife beating. Despite the different measurements of wife beating in two surveys we found almost similar prevalence and patterns of risk factors in both surveys. This similarity can be resulting from an increased reporting due to a change in attitudes or because of a more detailed questioning in the newer survey, paralleling a true decrease in the prevalence between the surveys. Formally, the Arabic translation of the question used in the 1995 survey covered almost all items used in the 2005 questionnaire [19], and the most frequently in 2005 reported items would qualify as beating, but some items might be less obvious. We therefore conducted additional analyses with a more restricted selection of items for the outcome variable in 2005 survey, which resulted in only a slight change in the reported prevalence. Still, a more detailed question used in 2005 may have prompted higher reporting than the single question used in 1995. While we are not aware of any study directly comparing the two approaches used in DHS surveys with respect to reporting of violence, there is some evidence that asking about violence in different sections of the questionnaire, using different wording and multidimensional measures improves reporting of violence [20,21]. While the issue of the extent of underreporting remains unresolved, there was also some indication of societal changes affecting the reporting of violence, which can be seen in the patterns of reporting and milder forms of violence experienced by better educated women. Differential reporting of violence across different characteristics of the respondents was observed in other studies [22] and additional dynamics can be introduced by changes in attitudes towards violence in the society.

Our analysis showed less pronounced risk factors associated with wife beating in the more recent EDHS and thus more homogeneity in wife beating across the strata of the population. This could be caused by a more general acceptance of wife beating in the population [12] or for example increased reporting of milder forms of violence in groups with lower prevalence.

When the education of both partners was evaluated jointly, it became clear that the woman's education made the difference with regard to wife beating. However, the rate of wife beating was reduced only when also the partner had secondary or higher education himself (the other case, when woman had a higher education than her partner, did not show a reduction in wife beating). Also the forms of wife beating differed by education level, a finding not reported from previous studies, but one that seems likely. A higher educational level of the respondent and her partner was found to be associated with a lower risk of violence also in the recent review by Jewkes [6]. On the contrary, most of the previous studies focused on education of the female partner only [23-26]. In a study in rural areas of Bangladesh, wife beating was reduced when women contributed economically to the household [23]. But the relationship between education of the women and wife beating is more complex: when women had a higher level of education than their partners, the levels of wife beating were higher in a study in Albania [27]. Communistic countries encouraged women's education, and women's rights were part of the political agenda [28]. In Turkey, women in higher education have career development perspectives which are partly better than in Western European countries [29]. But while dominant religion in Turkey is also Islam, there are strong cultural differences between Arabic Islamic countries and Turkey, and educated women might face more difficulties in Arabic Islamic countries [30]. Higher levels of violence experienced by educated women when their partner was less educated were also found in Kenya, with 50% of the population adhering to Islam [31]. In summary, it appears that raising education levels in the whole society, both men and women, is needed to reduce violence. When solely men are granted benefits of education, their violence behaviour might remain determined by patriarchal ideology and gender roles [32,33].

Youngest age group showed highest odds ratios of wife beating in both surveys. This is consistent with the findings of another study in Egypt [34]. Younger women have also short duration of marriage and young age at marriage, both factors which can contribute to higher prevalence of violence. In contrary, there was no effect of woman's age on prevalence of violence as reported by men in a study in Bangladesh [35]. In Bangladesh the prevalence of physical violence in the last 12 months was between 60 and 70% across all age groups, which is substantially higher than in our analysis and suggests a higher cultural acceptance of violence. Women having fewer children (adjusted for age) had lower risk of being beaten in our analysis. This might indicate less traditional cultural background and thus lower acceptance of violence among these women. This finding is consistent with the findings of other studies in Bangladesh [35] and Mexico [36].

## Conclusion

Beating by an intimate partner remains highly prevalent in Egypt, despite increasing levels of education in the population and initiatives to reduce wife beating. However, patterns of wife beating may have moved towards less severe forms in recent years. The woman's level of education appears a strong determinant of reduced wife beating, but only when her partner is also educated. Thus, improving women's access to education and raising levels of education in the whole society are promising strategies to reduce wife beating. Wife beating is all prevalent – not limited to selected risk groups – therefore interventions oriented towards the whole society rather than risk group oriented interventions are warranted. The concurrence of different trends and developments – like shifts in the level of education in the society or codes of behaviour within a social position – results in a very complex picture of societal changes. To allow an analysis of trends, studies should use the same and standardized instruments. Additionally, changes in reporting attitudes should be measured and potential changes in patterns of wife beating should be taken into consideration.

## Competing interests

The authors declare that they have no competing interests.

## Authors' contributions

MKA performed the analysis and drafted the manuscript. RTM contributed to the development of the research question, supervised the analysis and wrote the final version of the manuscript. SL developed the idea of the analysis and provided comments on the manuscript. ED and MMHK contributed to the discussion section. All authors read and approved the final manuscript.

## Pre-publication history

The pre-publication history for this paper can be accessed here:


